# Recent advances in preoperative management of esophageal adenocarcinoma

**DOI:** 10.12688/f1000research.10794.1

**Published:** 2017-04-18

**Authors:** Kazuto Harada, Dilsa Mizrak Kaya, Hideo Baba, Jaffer A. Ajani

**Affiliations:** 1 Department of Gastrointestinal Medical Oncology, University of Texas MD Anderson Cancer Center, Holcombe Boulevard, Texas, USA; 2 Department of Gastroenterological Surgery, Kumamoto University, Jonjo, Kumamoto, Japan

**Keywords:** Esophageal cancer, preoperative therapy, personalized therapy

## Abstract

Esophageal cancer is an aggressive malignancy with increasing incidence, and the prognosis of patients treated by surgery alone remains dismal. Preoperative treatment can modestly prolong overall survival. Preoperative chemotherapy or chemoradiation is the standard of care for resectable esophageal cancer (greater than clinical stage I and less than clinical stage IV). One of the challenges is to predict complete response in the surgical specimen from preoperative therapy and to avoid surgery in some patients but also predict ineffectiveness of preoperative therapy if the tumor is resistant and avoid such therapies altogether. In-depth understanding of the molecular biology could lead to personalized therapy, and in the future, clinical trials designed according to molecular features are expected. Here, we summarize preoperative treatment for esophageal adenocarcinoma and their potential.

## Introduction

Esophageal cancer (EC) is estimated to be the eighth most common cause of cancer in the world (456,000 cases) and the sixth most common cause of cancer death (400,000 deaths) ^1^. EC has two common histologic types: adenocarcinoma (EAC) and squamous cell carcinoma (ESCC). EAC is becoming prevalent worldwide, especially in North America and Western Europe ^2^. Esophagectomy is the most effective treatment for loco-regional control, but the 5-year survival rate after esophagectomy for locally advanced EC without preoperative treatment is less than 30% ^3^. For early-stage EC, endoscopical resection or esophagectomy without preoperative therapy is one of the available options ^4^. For the metastatic EAC, two-drug cytotoxic regimens, mainly a combination of a fluoropyrimidine and a platinum compound is recommended, and if EAC overexpresses HER2, trastuzumab should be added to chemotherapy ^4^. In case of locally advanced EC, despite radical resection, local-regional and distant recurrence develop in 33% and 20% of patients after resection, respectively ^5^. The pre-existing occult micrometastases or unresected occult local disease is responsible for relapses. Interestingly, in one study, at the time of operation, 88% of patients with EC were already found to have micro-metastases in rib marrow aspirated during esophagectomy ^6^. To overcome relapses after surgery, preoperative or postoperative treatments have been developed ^4^. Importantly, preoperative therapy can modestly prolong overall survival (OS) and increase the R0 resection rate. R0 resection is associated with a longer survival ^7^. Moreover, if preoperative therapy leads to a pathological complete response (pCR), longer OS may be expected ^8, 9^. Table 1 summarizes the preoperative therapy trials for EC conducted so far.

**Table 1.** Key esophageal cancer trials.

Study	Enrolled number	Treatment	Overall survival	Hazard ratio (95% confidence interval)	*P* value	References
Preoperative chemotherapy						
MRC OEO2	802	CF → surgery (n = 400) Surgery (n = 402)	5-year rate: 23% 5-year rate: 17%	0.84 (0.72-0.98)	0.03	11
FNCLCC and FFCD	224	CF → surgery (n = 113) Surgery (n = 111)	5-year rate: 38% 5-year rate: 24%	0.69 (0.50-0.95)	0.02	13
MAGIC	503	ECX → surgery → ECX (n = 250) Surgery (n = 253)	5-year rate: 36% 5-year rate: 23%	0.75 (0.60-0.93)	0.009	12
INT 113	440	CF → surgery (n = 213) Surgery (n = 227)	Median: 14 months Median: 16 months	1.04 (0.84-1.29)	0.53	15
MRC OEO5	897	ECF → surgery (n = 446) CF → surgery (n = 451)	3-year rate: 39% 3-year rate: 42%	0.92 (0.79-1.08)	0.30	14
Preoperative chemoradiotherapy						
CROSS	368	Taxol/carbo/41.4 Gy → surgery (n = 180) Surgery (n = 188)	Median: 48 months Median: 24 months	0.68 (0.53-0.88)	0.003	18
FFCD 9901	195	CF/45 Gy → surgery (n = 98) Surgery (n = 97)	5-year rate: 41% 5-year rate: 33%	0.99 (0.69-1.30)	0.94	21
CALGB 9781	56	CF/50.4 Gy → surgery (n = 30) Surgery (n = 26)	5-year rate: 39% 5-year rate: 16%	- (0.17-0.68)	0.002	20
Preoperative chemotherapy vs. preoperative chemoradiotherapy						
POET	119	CF/30 Gy → surgery (n = 60) CF → surgery (n = 59)	3-year rate: 47% 3-year rate: 27%	0.67 (0.47-1.07)	0.07	22
Burmeister *et al*.	75	CF/35 Gy → surgery (n = 39) CF → surgery (n = 36)	Median: 32 months Median: 29 months	-	0.83	23
NeoRes	181	CF/40 Gy → surgery (n = 91) CF → surgery (n = 90)	3-year rate: 47% 3-year rate: 49%	-	0.77	24

CALGB, Cancer and Leukemia Group B; CF, cisplatin and 5 fluorouracil; CROSS, Chemoradiotherapy for esophageal Cancer Followed by Surgery Study; ECF, epirubicin, cisplatin, and fluorouracil; ECX, epirubicin, cisplatin and capecitabine; FFCD, Fédération Francophone de Cancérologie Digestive; FNCLCC, Fédération Nationales des Centres de Lutte Contre le Cancer; MAGIC, Medical Research Council Adjuvant Gastric Infusional Chemotherapy; MRC, United Kingdom Medical Research Council; POET, Preoperative Chemotherapy or Radiochemotherapy in Esophago-gastric Adenocarcinoma.

## Preoperative chemotherapy

Several trials have produced mixed results. Firstly, the United Kingdom Medical Research Council (MRC) esophageal cancer trial (OEO2) recruited 802 patients (EAC: 67%) and randomly assigned them to two treatment groups: 400 to surgery plus perioperative chemotherapy—two cycles of FP (cisplatin and fluorouracil)—and 402 to surgery alone. As compared with the surgery group, the perioperative chemotherapy group had a favorable OS (5-year rate: 23% versus 17%; hazard ratio [HR] 0.84; 95% confidence interval [CI] 0.72 to 0.98; *P* = 0.03), thus showing only marginal benefit ^10, 11^. The other two trials added postoperative chemotherapy to preoperative chemotherapy. The Medical Research Council Adjuvant Gastric Infusional Chemotherapy (MAGIC) trial evaluated the effect of perioperative chemotherapy— three preoperative and three postoperative cycles of ECF (epirubicin, cisplatin, and fluorouracil)—for resectable gastro-esophageal (GE) adenocarcinoma ^12^. Five hundred and three patients were randomly assigned to two treatment groups: 250 to surgery plus perioperative chemotherapy and 253 to surgery alone. As compared with the surgery-alone group, the perioperative chemotherapy group had a favorable OS (5-year rate: 36% versus 23%; HR 0.75; 95% CI 0.60 to 0.93; *P* = 0.009). However, only 25% of patients in this trial had EAC or GE junction involvement. Finally, in the Fédération Nationales des Centres de Lutte Contre le Cancer/Fédération Francophone de Cancérologie Digestive (FNCLCC/FFCD) trial, 224 patients were randomly assigned to two treatment groups: 113 to surgery plus perioperative chemotherapy (two or three preoperative and three or four postoperative cycles of CF) and 111 to surgery alone ^13^. In this trial, 75% of patients had EAC. Compared with the surgery-alone group, the perioperative chemotherapy group had had a favorable OS (5-year rate: 38% versus 24%; HR 0.69; 95% CI 0.50 to 0.95; *P* = 0.02). Moreover, perioperative chemotherapy significantly increased the R0 resection rate (84% versus 73%; *P* = 0.04). These trial results were considered acceptable, and perioperative chemotherapy became standard therapy in Europe. Recently, the MRC-OEO5 trial compared two chemotherapy regimens: two cycles of FP and four cycles of ECX (epirubicin, cisplatin, and capecitabine) ^14^. The ECX group had a higher R0 resection rate and pCR; however, there was no OS benefit for ECF compared with FP (3-year rate: 42% versus 39%; HR 0.92; 95% CI 0.79 to 1.08; *P* = 0.30). Furthermore, chemotherapy toxicity was higher in the ECX group ^14^. This trial suggests that preoperative chemotherapy with more drugs and longer duration is not worthwhile and the addition of epirubicin does not provide any advantage.

The first and only study conducted in the US was the RTOG trial 8911 (USA Intergroup 113), which demonstrated no advantage from the addition of preoperative chemotherapy to surgery ^15, 16^. Thus, enthusiasm for preoperative chemotherapy has been low in the US and preoperative chemoradiation has been favored.

## Preoperative chemoradiation

A prior meta-analysis proposed that preoperative chemoradiation may be beneficial; however, meta-analyses are only hypothesis-generating ^17^. In 2012, the Chemoradiotherapy for esophageal Cancer Followed by Surgery Study (CROSS) trial produced favorable results for patients who received preoperative chemoradiation over surgery alone ^18^. Three hundred sixty-eight resectable but selected patients with EC (EAC: 75%) were randomly assigned to two treatment groups: 180 to surgery plus preoperative chemoradiation and 188 to surgery alone. The long-term result of the CROSS study showed that the median OS for the preoperative chemoradiation group was significantly longer than that for the surgery-alone group (median of 48.6 versus 24.0 months; HR 0.68; 95% CI 0.53 to 0.88; *P* = 0.003) ^19^. Importantly, the benefit for patients with ESCC was higher than for patients with EAC: Median OS rates for patients with ESCC were 81.6 months in the preoperative chemoradiation group and 21.1 months in the surgery-alone group (HR 0.48; 95% CI 0.28 to 0.83; *P* = 0.008), whereas the rates for patients with EAC were 43.2 months in the preoperative chemoradiation group and 27.1 months in the surgery-alone group (HR 0.73; 95% CI 0.55 to 0.98; *P* = 0.038) ^19^. The rate of R0 resection increased because of preoperative chemoradiation. A previous prospective randomized CALGB 9781 trial compared surgery plus preoperative chemoradiation (cisplatin and fluorouracil with 50.4-Gy concurrent radiotherapy) and surgery alone and showed benefit for preoperative chemoradiation ^20^. However, this trial assessed only 56 patients (EAC: 75%).

The benefit from preoperative chemoradiation for patients with early-stage EC remains debatable. The result of the FFCD 9901 trial, which compared the surgery-alone group (n = 97, EAC: 28%) with the preoperative chemoradiation group (n = 98, EAC: 31%), unfortunately did not show an increase in the R0 resection rate or OS benefit but did have an increase in postoperative mortality ^21^.

The type of chemotherapy agents and radiation dose differ among trials. The CROSS study used paclitaxel and carboplatin plus 41.4-Gy concurrent radiotherapy; on the other hand, the FFCD 9901 study used CF plus 45-Gy concurrent radiotherapy. Although the OS benefit from preoperative chemotherapy is modest to marginal, the types of combinations have varied (FP or ECF regimen) ^12, 13^. In the current National Comprehensive Cancer Network guidelines, ECF has been downgraded on the basis of the OEO5 trial results ^14^. Importantly, no trial has been completed that compared preoperative chemotherapy with preoperative chemoradiation. An ongoing trial might settle this issue. The PROTECT (PReoperative Chemoradiation (Paclitaxel-carboplatin or FOLFOX) for Resectable Esophageal and Junctional Cancer) study (NCT02359968) is comparing carboplatin and paclitaxel with FOLFOX during radiation. Another phase II trial (NCT01843829) is comparing carboplatin and paclitaxel with oxaliplatin and capecitabine. Additionally, the Neo-AEGIS trial (NCT01726452) is currently comparing the CROSS regimen with the MAGIC regimen. Results are expected in the near future.

## Chemotherapy and chemoradiation

A recent meta-analysis compared preoperative chemoradiotherapy (n = 1,078) and chemotherapy (n = 1,141) for both EAC and ESCC, showing better OS of preoperative chemoradiation but not to a significant degree (HR 0.88; 95% CI 0.76 to 1.01; *P* = 0.07) ^17^. To date, three randomized trials have compared preoperative chemoradiotherapy with chemotherapy, but none showed a benefit in OS of preoperative chemoradiation. The Preoperative Chemotherapy or Radiochemotherapy in Esophago-gastric Adenocarcinoma (POET) trial failed to recruit a sufficient number to document an OS advantage for preoperative chemoradiation, and the trial had to be terminated early ^22^. The pCR rate was higher with preoperative chemoradiation compared with preoperative chemotherapy (15.6% versus 2.0%) ^22^. Another phase II trial also showed no benefit from preoperative chemoradiation for patients with EAC; the median OS was 32 months from preoperative chemoradiation compared with 29 months from preoperative chemotherapy ( *P* = 0.83) ^23^. Another trial compared neoadjuvant chemotherapy with chemoradiotherapy in resectable cancer of the esophagus and gastric cardia patients (n = 181 with 73% EAC), and although chemoradiation resulted in a higher pCR rate (28% versus 9%), higher R0 resection rate (87% versus 74%), and a lower rate of lymph nodal metastases (35% versus 62%), there was no OS benefit (3-year OS: 49% versus 47%; *P* = 0.77) ^24^.

Our group retrospectively reviewed sequential phase II/III trials performed at the University of Texas M.D. Anderson Cancer Center, showing that compared with preoperative chemotherapy, preoperative chemoradiation exhibited a longer OS ( *P* = 0.046) and disease-free survival ( *P* = 0.015) and the higher pCR rate ( *P* <0.001) ^25^.

## Induction chemotherapy followed by preoperative chemoradiation

Our group had proposed a strategy of induction chemotherapy before preoperative chemoradiation ^26^. To document whether there is any benefit to the addition of induction chemotherapy, we reported a randomized phase II trial that compared induction chemotherapy followed by preoperative chemoradiation with preoperative chemoradiation only. One hundred twenty-six patients with localized EAC were randomly assigned to one of two groups. The median OS rates with and without induction chemotherapy were 43.6 and 45.6 months, respectively. The pCR rates were 13% in the no induction chemotherapy group and 26% in the induction chemotherapy group ( *P* = 0.094), concluding that induction chemotherapy did not appear to benefit these patients ^27^. However, subgroup analysis showed that induction chemotherapy had a considerable benefit for only those patients who had a well-moderate differentiated tumor ^28^.

## Preoperative treatment with molecular targeting drug

Currently, there is no evidence that the addition of a targeted drug benefits to patients with localized EC. In patients with advanced EAC or gastric cancer, the ToGA (Trastuzumab for Gastric Cancer) trial showed that the addition of HER2 inhibitor, trastuzumab, to chemotherapy had modest benefit ^29^. However, the benefit of trastuzumab in the neoadjuvant setting is not established. In Japan, a phase II trial is evaluating S-1 plus cisplatin with or without trastuzumab in the neoadjuvant setting for HER2-positive gastric or esophagogastric junction adenocarcinoma ^30^. Epidermal growth factor receptor (EGFR) inhibitors have been evaluated in this setting ^31– 34^ on the basis of a tantalizing phase II study which added cetuximab to chemoradiation and produced a pCR rate of 27% ^33^. However, two phase III trials that added an EGFR inhibitor to dCRT (definitive chemoradiation) failed to show survival benefit ^31, 35^. In addition, bevacizumab or erlotinib was evaluated with preoperative chemoradiation but did not demonstrate survival benefit or improvement in the pCR rate ^36^.

## Future perspective

Approximately 25% of patients who undergo preoperative chemoradiation achieve a pCR ^18^. If one could predict the possibility of pCR with a high level of certainty, then novel strategies to preserve the esophagus could be implemented. However, there are no useful clinical variables including positron emission tomographic (PET) changes and there are no reliable biomarkers for such a prediction at the moment. A clinical CR defined as endoscopic biopsies without cancer cells and PET scan with physiologic uptake provides an OS benefit ^37^ but does not correlate with pCR ^38^. Therefore, we recommend that all patients eligible for surgery proceed to surgery after recovering from chemoradiation.

Metabolic imaging with 18-fluorodeoxy-glucose positron emission computed tomography was assessed in various circumstances. Our group reported that PET parameters could correlate with prolonged OS but could not predict pCR ^39^. Interestingly, the Municon I and II trials evaluated treatment modifications according to PET responses for patients with EAC ^40, 41^. In the Municon II trial, PET non-responders from preoperative chemotherapy had chemoradiation before surgery; however, non-responders still had an unfavorable prognosis, suggesting that primary resistance cannot be overcome by simply changing therapy. Thus, in-depth study of the tumor biology is warranted and may contribute to personalize therapy.

Recently, whole-genome analyses of EAC have been reported ^42– 45^. The Cancer Genome Atlas reported an integrated genomic landscape in EC, showing that genomic characterization of EAC was different from that of ESCC but similar to that of gastric cancer subtype “CIN, chromosomal instability” ^46, 47^. Mutations in *TP53*, *CDKN2A*, *ARID1A*, and *SMAD4* were common in EAC. Amplifications in *ERBB2*, *VEGFA*, *GATA4*, and *GATA6* were common in EAC. A positive result for microsatellite instability or Epstein-Barr virus was rare in EAC. However, there were some differences between EAC and gastric cancer subtyped with CIN. Compared with gastric cancer, EAC had more frequent CpG hyper-methylation phenotype, *VEGFA* and *MYC* amplifications and mutation of *SMARCA4*, deletion of tumor suppressor *RUNX1*, *FHIT*, and *WWOX*, and lower *APC* pathway activation ^46^. The molecular features of EAC were significantly different from that of ESCC. ESCC had significantly mutated genes, such as *TP53*, *NFE2L2*, *MLL2*, *ZNF750*, *NOTCH1*, and *TGFBR2*, and specific somatic copy number alterations, such as amplifications of SOX2, TERT, FGFR1, MDM2, and NKX2-1 and deletion of RB1. Compared with EAC, inactivation of CDKN2A, amplification of CCND1 and TP63/SOX2, and alterations of histone-modifying factors were more common in ESCC; conversely, ERBB2 alterations were rare, suggesting that molecular targeting can differ between EAC and ESCC ^46^. Secrier *et al*. reported whole-genome sequencing in 129 EAC samples and classified EAC into three groups: C>A/T dominant (29%), DNA damage repair (DDR) impaired (18%), and mutagenic (53%) ^45^. The report recommends that, in some patients, the combination of anti-ERBB2 and anti-MET inhibition might prove useful. In the presence of DDR impairment, inhibition by poly ADP ribose polymerase (PARP) inhibitor in combination with DNA-damaging agent might prove useful. Recently, immune-checkpoint pathways, such as T lymphocyte-associated antigen 4 (CTLA-4) and programmed death protein 1/its ligand (PD-1/PD-L1), have received much attention ^48^. Therefore, tumors that have high mutation load may be amenable to immune-checkpoint inhibitors ^49^.

Several biomarkers that may be associated with response to preoperative therapy have been explored ^50^. For instance, 3′-untranslated region polymorphisms of thymidylate synthase may predict a response to 5-fluorouracil (5-FU)-based chemoradiation ^51^. Overexpression of excision repair cross-complementation group 1 may be associated with chemoradiation response, especially with platinum agent ^52^. MicroRNAs also have potential as predictive markers ^53^. Hale *et al*. reported that the proportion of tumor in biopsy tissue can predict preoperative chemotherapy response ^54^; however, these data need to be validated and combined with biomarkers.

Recently, liquid biopsy has been actively studied and is of considerable interest ^55^. Tumor-derived biomarkers in the bloodstream, such as circulating tumor cells (CTCs), cell-free DNA (ctDNA), and exosomes, have the potential to predict early treatment response ^56, 57^. For instance, in several cancers, changes in CTC count were associated with response to treatment ^58, 59^. In colorectal cancer, during treatment, mutation or copy number status in ctDNA can be dynamically monitored ^60, 61^. Further studies and clinical applications are expected.

## Conclusions

This review described an understanding of preoperative therapy for EC. The benefits of preoperative treatment, and preoperative chemoradiation in particular, have been established. Currently, preoperative chemoradiation is preferred over preoperative chemotherapy in the US. Head-to-head comparison of preoperative chemotherapy versus preoperative chemoradiation is not completed as it is a subject of ongoing trials. A further challenge is to identify patients who are destined to achieve a pCR. CTCs or ctDNA might prove useful in surveillance after therapy and occasionally for selection of therapy.
